# Rapid Authentication of Potato Chip Oil by Vibrational Spectroscopy Combined with Pattern Recognition Analysis

**DOI:** 10.3390/foods10010042

**Published:** 2020-12-25

**Authors:** Siyu Yao, Didem Peren Aykas, Luis Rodriguez-Saona

**Affiliations:** 1 Department of Food Science and Technology, The Ohio State University, 110 Parker Food Science and Technology Building, 2015 Fyffe Road, Columbus, OH 43210, USA; yao.806@osu.edu (S.Y.); aykas.1@osu.edu (D.P.A.); 2 Department of Food Engineering, Faculty of Engineering, Adnan Menderes University, Aydin 09100, Turkey

**Keywords:** rapid authentication, handheld Raman, NIR, fatty acid profile, oil qualification

## Abstract

The objective of this study was to develop a rapid technique to authenticate potato chip frying oils using vibrational spectroscopy signatures in combination with pattern recognition analysis. Potato chip samples (*n* = 118) were collected from local grocery stores, and the oil was extracted by a hydraulic press and characterized by fatty acid profile determined by gas chromatography equipped with a flame ionization detector (GC-FID). Spectral data was collected by a handheld Raman system (1064 nm) and a miniature near-infrared (NIR) sensor, further being analyzed by SIMCA (Soft Independent Model of Class Analogies) and PLSR (Partial Least Square Regression) to develop classification algorithms and predict the fatty acid profile. Supervised classification by SIMCA predicted the samples with a 100% sensitivity based on the validation data. The PLSR showed a strong correlation (Rval > 0.97) and a low standard error of prediction (SEP = 1.08–3.55%) for palmitic acid, oleic acid, and linoleic acid. 11% of potato chips (*n* = 13) indicated a single oil in the label with a mislabeling problem. Our data supported that the new generation of portable vibrational spectroscopy devices provided an effective tool for rapid in-situ identification of oil type of potato chips in the market and for surveillance of accurate labeling of the products.

## 1. Introduction

The potato chip was invented 167 years ago and has been the most popular snack food in America for more than 50 years [1,2]. Oil represents between 25% and 35% weight of the potato chip, serving as the heat transfer agent and providing the flavor and texture of the product [3]. As reported by researchers, the main precursors of volatile compounds in potato chips are polyunsaturated fatty acids in the frying oil [4,5,6]. The non-heterogeneous oil distribution during the frying contributes to the surface color of potato chips [7]. The common types of oil utilized in potato chip manufacturing are corn, sunflower (mid-oleic and high-oleic varieties), canola, high-oleic (HO) safflower, and cottonseed oils [8].

As the trend toward wellness keeps gaining strength, the selection of oils can add value as healthier alternatives. For example, systematic studies suggested that consuming foods rich in monounsaturated or polyunsaturated fat positively affected blood glucose control, compared with consuming saturated fat or dietary carbohydrate, and may help to prevent metabolic diseases [9,10]. Accordingly, numerous potato chip manufacturers are selecting oils with high-oleic traits to meet buyer healthier preferences. However, adulteration of high-price oils is a prevalent source of economically-motivated fraud [11]. Canola, soybean, and palm oils become common adulterants for high price oils like sunflower oil, which has a higher content of unsaturated fatty acid [12]. Therefore, there is an urgent need for authentication and prevention of adulteration for the sake of consumers and honest companies.

Accurate and appropriate analytical methods are required to identify the oil type based on their components [13,14]. Traditionally, fatty acid methyl esters (FAMEs) are analyzed by gas chromatography with flame ionization detector (GC-FID) to determine oil types based on the fatty acid composition, and Iodine value (IV) is utilized to classify oils according to their degree of unsaturation [15,16,17]. However, these conventional methods are labor-intensive, time-consuming, high-priced, require the use of harmful reagents and generate hazardous waste [18]. Hence, it is necessary to develop technologies that can provide real-time screening and in-field applications to authenticate the oil used in potato chip manufacturing. Vibrational spectroscopy (near-infrared (NIR), mid-infrared (mid-IR) and Raman) are rapid methods to offer a high throughput, simple, sensitive and robust technique for establishing reliable authentication for raw materials, based on their specific signature profiles coupled with pattern recognition techniques [19].

Raman spectroscopy (50–8000 cm^−1^) is based on the inelastic scattering of monochromatic light [20,21]. When the sample interacts with the monochromatic laser, in addition to the relatively more pronounced elastic scattering effect in the mode of Rayleigh scattering, an inelastic scattering can arise which results in new photon emissions with different frequencies or a shift from that of the excitation light. This scattering is called Raman scattering, whereby Raman shifts are directly related to the vibrational states of a molecule structure [22]. Near-infrared (NIR) spectroscopy (800–2500 nm) is based on molecular overtone and combination vibrations in the region of the electromagnetic spectrum [23]. For a molecule to be Raman active, the polarizability of the molecule needs to be changed through incident radiation and a center of symmetry is required, while for NIR activity to be dominant, the dipole moment of the molecule has to be changed and, thus, the molecule ought not have a center of symmetry. Therefore, usually the molecules which are Raman active are not IR active and vice versa [24].

Meanwhile, advancement in semiconductors has allowed the miniaturization of the components such as solid-state lasers, wavelength selectors, and detectors leading to commercially accessible and affordable portable, handheld, compact, and micro-vibrational spectroscopy devices in the industry [19]. These portable/handheld spectrometers have the tremendous potential capability to move from the confines of the comparatively steady and controlled laboratory setting to the potentially more dynamic and complex environments at- or in-line, at points of vulnerability along complicated food supply chains [25].

However, limited information is reported in the literature regarding the rapid authentication of oils used in manufacturing potato chips using vibrational spectroscopy. Aykas et al. [8] evaluated a portable MIR in conjunction with pattern recognition analysis to develop classification methods for the authentication of potato chip oils. Nonetheless, the measurement process needs heating for preventing oil solidification, which limits the in-field application. Baeten et al. [26] assessed the oil and fat classification by Raman spectroscopy (1064 nm) by using principal component analysis (PCA) that was applied to 138 samples from 21 different sources and reported that stepwise linear discriminant analysis can classify oils based on their unique monounsaturated and polyunsaturated composition. Dong et al. [27] established a predictive model of the fatty acid composition of vegetable oil based on least squares support vector machines (LS-SVM), by Raman (785 nm) spectral data. McDowell et al. [24] built calibration models with four different multivariate classifiers (soft independent modeling of class analogy (SIMCA), linear discriminant analysis—k-nearest neighbor (LDA-KNN), partial least squares—discriminant analysis (PLS-DA), and linear discriminant analysis—support vector machine (LDA-SVM)) based on either FT-IR and Raman spectral fingerprints to detect the oil addition in cold-pressed rapeseed, achieving high sensitivity of 86% and 93%, respectively, when refined sunflower oil is the adulterant. These studies have shown the potential capabilities of vibrational spectroscopy to detect vegetable oil adulteration. However, they do not show sufficient ability to classify based on different types of vegetable oils, and they have not applied the analysis to oil expelled from the real food matrix. Moreover, most have been developed using a limited number of oil types, limiting their application as a reliable method to detect oil adulteration of food products in the market [28].

The objective of this study was to develop a rapid detection method to identify the type of oil used in the manufacturing of potato chips and to predict the fatty acid profile of the oil based on the unique Raman and NIR spectral patterns.

## 2. Materials and Methods

A total of 118 potato chip samples, including 102 samples for generating the training models and 16 samples serving as an independent external validation set, were collected from local grocery stores in Columbus, OH. The potato chips (~10 g) were pressed to expel oil (~3 g) by a manual hydraulic press (3851 Benchtop Laboratory Manual Press, Carver, Inc., Wabash, IN, USA). The crushed potato chips filled a stainless-steel cylinder container. The oil was expelled by applying pressure on the cylinder to 10,000 psi for 1 min. Oil is collected and stored at 3 °C in the glass vials for further analysis. Six different reference vegetable oils, including corn, canola, sunflower (high-oleic and mid-oleic), peanut, and cottonseed oils, were collected from online vendors and local stores.

### 2.1. Reference Method

The reference method for obtaining the fatty acid profile is based on a fatty acid methyl ester (FAME) procedure with modification [29]. Methyl ester structures were produced by dissolving 100 μL oil sample with 1 mL of hexane into a 2 mL centrifuge tube, and the mixture was vortexed. Then 20 μL 2 N potassium hydroxide in methanol was added to the centrifuge tube and vortexed for 1 min. The upper hexane part was transferred to a new 2 mL centrifuge tube with one pinch of sodium sulfate anhydrous and centrifuged at 4000 rpm for 10 min. After that, 500 μL supernatant was transferred into a 2 mL GC glass vial and mixed with 700 μL hexane thoroughly for further analysis. FAME profile analysis was done in duplicate for all samples by an Agilent 6890 arrangement (Agilent Technologies, Inc., Santa Clara, CA, USA) gas chromatograph (GC) equipped with a flame ionization detector (FID), an Agilent 7693 autosampler (Agilent Technologies, Inc., Santa Clara, CA, USA), and a tray. The fatty acids were separated by utilizing an HP-88 60 m × 0.25 mm × 0.2 mm (Agilent 112-8867, Agilent Technologies, Inc., Santa Clara, CA, USA)) GC column and utilizing helium as the carrier gas. The injection volume was 0.1 μL, with a split ratio of 60.3: 1. The inlet and detector temperatures were 250 °C. The oven temperature was set at 120 °C held for 1 min as the initial, then at 175 °C (10 °C/min) held for 10 min, then at 210 °C (4 °C/min) held for 4 min and finally at 230 °C (4 °C/min) held for 4.75 min. Based on the reference standards (Supelco^®^ 37 Component FAME Mix, Sigma Aldrich, Inc., St. Louis, MO, USA), through the comparison of each peak’s retention times, fatty acids were identified [28]. All the samples (*n* = 118) were analyzed by GC-FID, and if the fatty acid composition of the sample matched with the profiles of reference oils or literature values, this sample was identified as being fried by the corresponding single oil source; otherwise, it was determined as being fried using oil mixtures.

### 2.2. Spectral Data Acquisition

#### 2.2.1. Raman Spectral Data Acquisition

**Figure 1:**
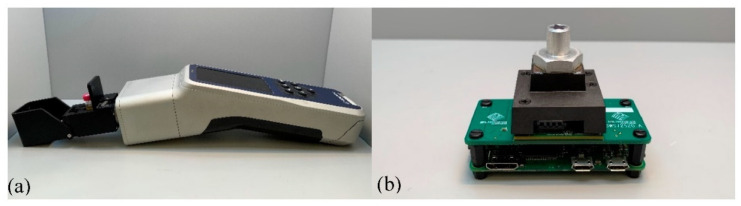
Potato chip oil spectrum acquisition by (**a**) using a handheld Raman instrument equipped with a 1064 nm excitation laser and by (**b**) using a compact Fourier Transform Near-Infrared (FT-NIR) spectral sensor.

A handheld Raman instrument, Progeny^TM^ (Rigaku Analytical Devices, Inc., Wilmington, MA, USA) equipped with a 1064 nm excitation laser (Figure 1a), was used to analyze the oil (at least 500 μL required) in the transparent glass vial obtained from the pressing process. The Raman device equipped with a thermoelectrically cooled InGaAs 512-pixel detector operated at 8 cm^–1^ spectral resolution with a spectral range of 200–2500 cm^–1^ [30]. The laser power and exposure time were set at 230 mW and 3 s, respectively, with 15 averages to maximize the signal-to-noise ratio. A background was collected after the spectrum was collected for each sample. The spectra were collected in duplicate for all samples (*n* = 118).

#### 2.2.2. NIR Spectral Data Acquisition

The NIR spectral data was collected by the NeoSpectra Micro (Si-Ware Systems, Inc., Cairo, Egypt), which is a compact Fourier Transform Near-Infrared (FT-NIR) spectral sensor with a single uncooled InGaAs photodetector utilizing a single-chip Michelson interferometer with monolithic opto-electro-mechanical structure based on Fourier Transform Infrared (FT-IR) technology [31]. A 100 μL oil aliquot was deposited onto the sensor of the spectrometer and the oil was covered with a reflectance accessory, NIRA Liquids Sample Accessory (Perkin Emerto, Inc., Llantrisant, Pontyclun, UK) to perform the measurement as shown in Figure 1b. An oil spectrum was collected in duplicate for all samples (*n* = 118) over the range of 1350–2552 nm in absorbance mode and a resolution of 25 cm^–1^. To get the best reproducibility and signal-to-noise ratio, the scanning time was set to 20 s.

### 2.3. Multivariate Data Analysis

The spectral data were analyzed by multivariate statistical analysis software, Pirouette^®^ (version 4.5, Infometrix, Inc., Bothell, WA, USA). Raman spectral data was transformed by normalization (sample 2-norm), where each data value was divided by the sample’s maximum value for SIMCA and PLSR analysis. NIR spectra were pre-processed by auto-scaling to correct for different scaling and units, and transformed by Savitsky–Golay second derivative (15 points with second-order polynomial filter) and Smoothing (to help reduce baseline noise) in the NIR SIMCA analysis. In the Raman and NIR PLSR analysis, mean-centering was utilized as the preprocessing method to alleviate “micro” but not “macro” multicollinearity [32].

The classification algorithm of potato chip oil was generated using the SIMCA method, a supervised classification method that clusters oil samples with common Raman or near-infrared spectral features and distinguishes them into their vegetable oil sources with different profiles based on principal component analysis (PCA) [33]. Samples were divided into training (83 single vegetable oil source samples verified by their FAME assignments) and external validation (16 samples, single oil and oil mixture samples) sets. The training set is utilized to “teach” the system about the Raman and NIR spectral features of each population (class) to determine whether discrimination differences are present, which is accomplished by providing the model with the class assignments based on GC-FID data. External validation of the SIMCA model’s performance was evaluated by an unseen independent dataset (16 samples) using the trained model, generating an unbiased estimation of the resembling model deployment for predictions in a real situation and determining if these potato chip oils match their “market” labels [34]. SIMCA model performance was evaluated in terms of misclassifications (percentage of samples correctly assigned to their original groups), class projections, discriminating power (most significant regions or wavenumbers for class separations), and interclass distances (ICD) describing the similarity or dissimilarity of the different classes quantitatively, it being accepted generally that samples can be well-differentiated when ICD > 3 [35].

PLSR is a quantitative technique for generating quantitative training predictive models through combining characteristics from multiple linear regression and PCA [30]. Raman and NIR spectra of all 102 samples (single oil source and oil mixture samples) were correlated with their fatty acid profile for developing PLSR predictive models. The performance of PLSR models for predicting fatty acid compositions were evaluated using leave-one-out as the internal cross-validation and an unseen independent dataset (16 samples) was set to validate the models externally. PLSR model performance was evaluated in terms of correlation coefficients (*R*^2^), residual analysis, outlier diagnostics, leverage, standard error cross-validation (SECV), and the standard error of prediction (SEP) [8]. If the leverage and/or studentized residual is high for a sample, this sample has a high possibility to be an outlier, and it was excluded from the model [28].

## 3. Results and Discussion

### 3.1. Characterization of Potato Chip Frying Oil (Fatty Acid Composition and Spectral Analysis)

To generate a training model for identifying the oil type used in the manufacturing, all the oils extracted from the potato chip samples were profiled based on the GC-FID method. Among all the samples (*n* = 102), based on their fatty acid profiles, 19 samples were identified as being fried using oil mixtures, while 83 samples were manufactured with a single vegetable oil source. The fatty acid compositions (C16:0, C18:0, C18:1 n-9, C18:2 n-6 and C18:3 n-3) of samples with single oil source were summarized in Table 1, including corn oil (*n* = 22), canola oil (*n* = 8), mid-oleic sunflower oil (*n* = 14), high-oleic sunflower oil (I) (*n* = 14), high-oleic sunflower oil (II) (*n* = 16), peanut oil (*n* = 4), and cottonseed oil (*n* = 5). Overall, cottonseed oil (17.6–21.8%) and corn oil (8.4–14.1%) showed the highest content of palmitic acid, while HO sunflower (I) oil (82.0–87.1%) showed the highest content of oleic acid, and cottonseed (57.0–59.1%) and corn oil (54.5–58.5%) showed the highest content of linoleic acid (Table 1).

To confirm the accuracy of oil type identification, fatty acid composition of oil from potato chip samples was compared with reference oils (Table 1) and literature values. The fatty acid profiles of corn, canola, high-oleic sunflower (I), mid-oleic sunflower and cottonseed oils were in agreement with our reference oils, and those reported by Caballero et al., Aykas et al., and Dubois et al. [8,36,37,38]. The peanut oil extracted from potato chip had a higher content of oleic acid (75.6–81.4%) and a lower content of linoleic acid (11.4–15.4%), compared to the values these researchers reported (around 52.1% and 32.9%, respectively). However, their fatty acid values fell into the fatty acid composition range (oleic acid: 52.8–82.2%, linoleic acid: 2.9–27.1%) found by Worthington et al. [39] for most cultivated peanuts. The discrepancies in the fatty acid composition under the same oil source can be related to differences in geographic origin and variety of seed-cultivars, and in seed and oil processes [40]. Interestingly, in the case of sunflower oil, three different fatty acid profiles (MO sunflower, HO sunflower (I) and HO sunflower (II)) were found. Stability of oil is directly related to its degree of unsaturation, and HO sunflower oils, which have over 70% oleic acid, are more stable than their counterparts with higher content of polyunsaturated fatty acids, linoleic and linolenic acids, fulfilling a better performance in the heating tolerance for a longer fry life [41,42,43]. The varieties of HO sunflower (I) oil containing over 80% oleic acid and HO sunflower (II) oil containing from 70% to 80% oleic acid can come from genetic selection, naturally occurring variation and trough mutagenesis [44].

**Figure 2:**
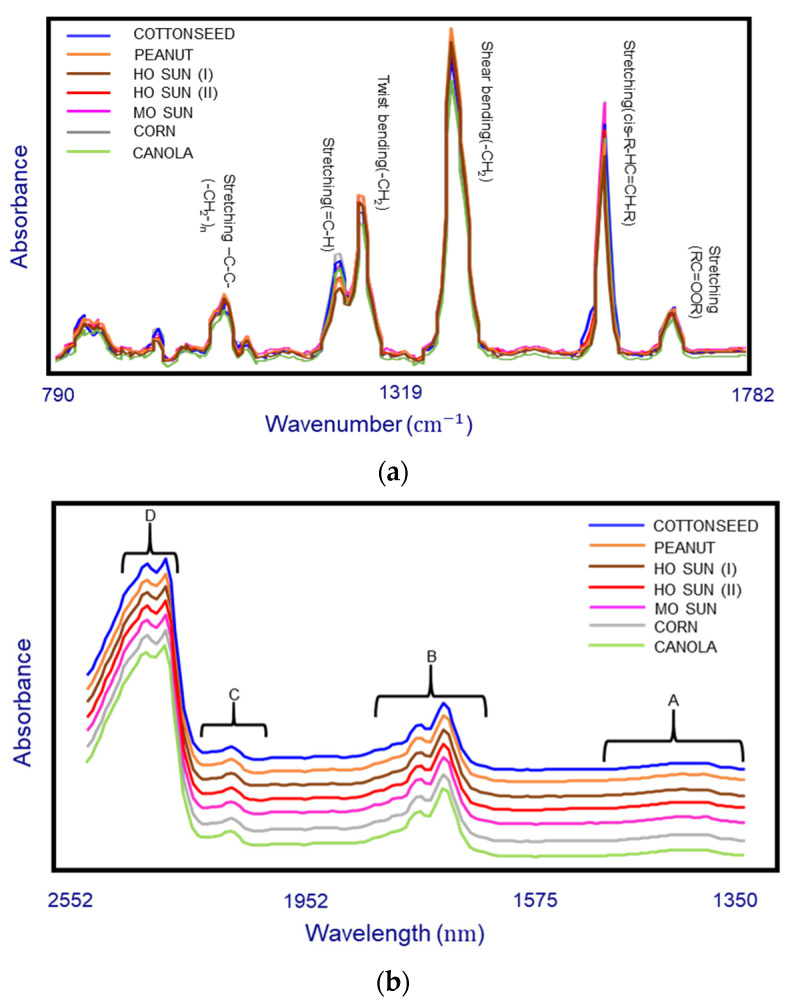
(**a**) Raman spectra and band assignments of some vegetable oil examples collected by a handheld Raman instrument equipped with a 1064 nm excitation laser. (**b**) Near-infrared (NIR) spectra and important absorbance regions of vegetable oils collected by a miniature NIR sensor.

Figure 2a showed the overlapped Raman spectra of seven different potato chip oils (cottonseed, peanut, HO sunflower (I), HO sunflower (II), MO sunflower, canola, and corn oils) and the corresponding band assignments. The band existing at 1745 cm^–1^ was the stretching vibration of ester bond carbonyl. The band at 1659 cm^−1^ was associated with C=C stretching (cis-R-HC=CH-R) from polyunsaturated fatty acids, while the band at 1263 cm^–1^ corresponds with in-plane =C-H deformation in an unconjugated cis (C=C), which was associated with monounsaturated fatty acids. The band at 1443 cm^–1^ was associated with CH_2_ scissoring deformation (δCH_2_), and the band at 1300 cm^–1^ was related to in-phase methylene twisting motion. The band at 1080 cm^–1^ was associated with the stretching vibration of the methylene chain skeleton [28,45]. As can be seen in Figure 2a, the signal to noise ratio was excellent across the spectral region and the Raman spectra patterns for these oils were similar to each other, but they appear to show an obviously different intensity on the bands of stretching (cis-R-HC=CH-R), shear bending (-CH_2_) and stretching (=C-H). An increase in the stretching (cis-R-HC=CH-R) and stretching (=C-H) bands intensity is correlated to the increase of unsaturated fatty acids weight percentage in oils [46], while the ratio of stretching (cis-R-HC=CH-R) to shear bending(-CH_2_) is inversely correlated with the content of saturated fatty acid [47].

Figure 2b showed the characteristic NIR absorption spectra of the seven different potato chip oil examples demonstrating the close similarity in their spectral characteristics. The peaks in NIR spectra were much broader compared with Raman. Briefly, characteristic NIR absorbance bands arise in four regions in the spectrum. Region A (1350–1490 nm) results from the combinations of C-H stretching and bending. Region B (1640–1885 nm) corresponds with the first overtone of the C-H stretching vibration of several chemical groups (methyl, methylene and ethylene groups). Furthermore, Region C (2050–2230 nm) is related to the C–H vibration of cis-unsaturation, and the intensity increasing in this region reflects the increase in the degree of total unsaturation. The two peaks in attributed fat could be observed clearly in the region D (2310–2350 nm), which represents the characteristic of the combination of C-H stretching vibration and other vibrational modes [48,49,50].

### 3.2. Pattern Recognition Modeling for Raman and NIR Spectroscopy

**Figure 3:**
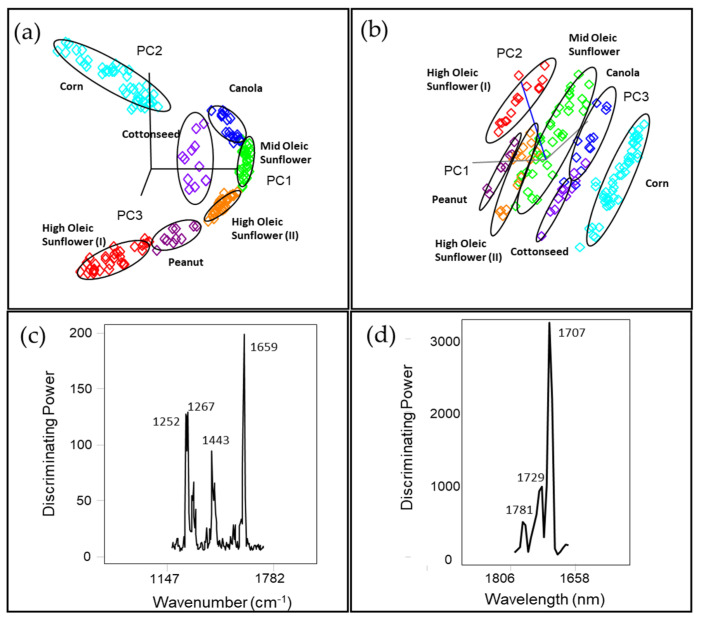
Soft Independent Model of Class Analogies (SIMCA) 3D projection plots of spectral data for potato chip oil samples collected by (**a**) a handheld Raman spectrometer (1064 nm) and (**b**) a miniature near Infrared (NIR) sensor. (**c**) Discriminating plots of Raman and (**d**) NIR SIMCA models showing bands and regions responsible for class separation.

The Raman and NIR spectral data were analyzed using SIMCA for the classification and rapid authentication of different frying potato chip oils based on the FAME profile. The class projection plot of the training SIMCA model generated with Raman spectral data (Figure 3a) showed distinctive clustering patterns and seven well-defined groups for different sole source oils in the three-dimensional (3D) environment. The interclass distances (ICD) shown in Table 2a describes the similarity or dissimilarity of the different classes quantitatively, ranging from 0.9 (MO SUN and HO SUN(II)) to 10.1 (HO SUN(I) and Corn) and it is generally accepted that samples can be differentiated when ICD > 3 [51]. Most of the classes, such as HO SUN(I) and MO SUN, HO SUN(I) and Canola Oil, HO SUN(I) and Corn oil, etc., are significantly differentiated between each other (ICD > 3), while some classes HO SUN(I) and HO SUN(II), HO SUN(I) and Peanut, MO SUN and Canola, MO SUN and HO SUN(II), HO SUN(II) and Peanut, and Corn and Cottonseed gave ICD < 3 because of the limited compositional difference among them [8]. In order to discriminate between the classes and minimize the overfitting problem, five principal components were employed to explain 99% of the variance. The discriminating power graph (Figure 3c) in the SIMCA model defines the variables (wavenumbers) mainly responsible for the potato chip oil classification [33], which can be representative of specific chemical structures. The band centered at 1659 cm^–1^ was associated with (cis-R-HC=CH-R) from polyunsaturated fatty acids, which has the most significant influence on classifying the samples. The band at 1443 cm^–1^ corresponded to the CH_2_ scissoring deformation, and bands at 1252 and 1267 cm^–1^ were related to stretching(=C-H), monounsaturated fatty acids.

The class projection of the SIMCA model generated by NIR spectral data (Figure 3b) showed similar grouping patterns obtained from Raman, but it improved class separation with larger interclass distances, yielding well-defined clusters using three to five principal components. There was no misclassification under the cross-validation and the interclass distances (Table 2b) among different classes of samples varying between 2.9 and 44.8. The highest ICD (44.8) was between HO SUN(I) and Canola oil, while there was only one group of classes that had an ICD < 3, which was between MO SUN and HO SUN(II). The SIMCA discriminating plot (Figure 3d) illustrated that the clustering of different potato chip oils was explained by the wavelength associated with 1707, 1729, and 1781 nm, corresponding to the first overtone of the C-H stretching vibration of several chemical groups (methyl, methylene, and ethylene groups).

**Figure 4:**
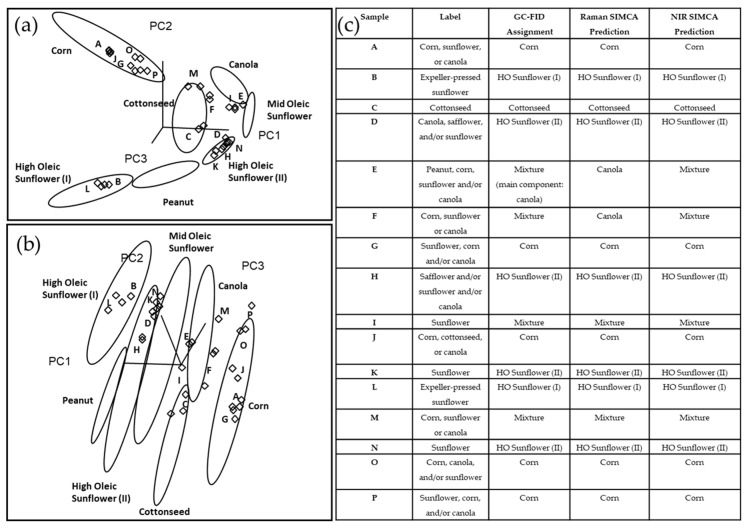
(**a**) Raman and (**b**) NIR SIMCA projection for the external validation set (*n* = 16). (**c**) Information summary of manufacture’s label claims, GC-FID assignments, Raman SIMCA predictions and NIR SIMCA predictions for the external validation set.

The predictive accuracy of SIMCA training models generated by the Raman and NIR spectral data was evaluated using an independent external validation set that included 16 commercial potato chip samples. Among them, only six samples were labeled with a single oil as their frying sources, including cottonseed, sunflower and expeller-pressed sunflower oils, and the remaining (*n* = 10) were labeled as having one or more type of oils. Figure 4a,b showed the Raman and NIR SIMCA 3D projection for the external validation set, respectively. Figure 4c summarized their label information, GC-FID analysis results, and Raman and NIR SIMCA predictions. Our GC-FID results showed that 12 out of 16 samples were manufactured with one type of vegetable oil, including corn, HO SUN(I), HO SUN(II) and cottonseed oils. Our Raman and NIR SIMCA predictions were consistent with the GC-FID assignments for all these 12 samples. Besides, 4 samples (E, F, I and M) were identified as having oil mixtures (two or more types of oils) based on their fatty acid profiles. SIMCA predictions of both Raman and NIR instruments indicated Sample I fried with oil mixtures and the GC-FID assignment confirmed; however, its label falsely indicated it as containing only sunflower oil. GC-FID assignment showed that sample E contained canola oil as its main component and at least one other type of oil. In the Raman and NIR SIMCA projection plots, Sample E was clustered close to canola and MO SUN classes in the 3D environment. Sample E was predicted as a mixture accurately in the NIR SIMCA prediction. However, due to the small interclass distance (1.5) between canola and MO SUN classes in the Raman SIMCA model, the oil from sample E was predicted as canola oil instead of the oil mixture in the Raman SIMCA prediction. The oil from Sample F was identified as a mixture based on its GC-FID result. In the Raman SIMCA projection plot, this oil mixture was clustered very close to the canola group, which led to the false prediction as canola oil. On the other hand, the NIR SIMCA model accurately predicted sample F as the oil mixture, though this sample was clustered close to the canola group in the NIR projection. Our results demonstrated some compositional similarities between canola oil and sample E and F. Sample M was also identified as an oil mixture based on GC-FID, and it was projected in the space closed to canola and corn clusters in the Raman and NIR projection plots. Raman and NIR SIMCA models both predicted sample M accurately as an oil mixture.

Sensitivity determined the ability of the classification model to identify the sole oil type of potato chips, while specificity evaluated the capability of our model to discriminate the oil mixture from the sole oil types [28]. The predictive performance statistics of the NIR SIMCA model showed 100% sensitivity (n_true positive_ = 12, n_false negative_ = 0) and 100% specificity (n_false positive_ = 0, n_true negative_ = 4) (Table 3) in classifying the independent samples, matching the results obtained from the GC-FID method. The Raman SIMCA model showed 100% sensitivity (n_true positive_ = 12, n_false negative_ = 0) and 50% specificity (n_false positive_ = 2, n_true negative_ =2) (Table 3) since Sample E and F which are oil mixtures based on the GC-FID results falsely predicted as samples using a sole oil source.

Similar to our research using the Raman approach, Yang et al. [50] used linear discriminant analysis (LDA) and canonical variate analysis (CVA) to discriminate corn oil, peanut oil, canola oil, safflower oil, etc., resulting in about 94% classification accuracy with their FT-Raman equipment. In addition, Velioglu et al. [52] differentiated seven vegetable oils successfully using principal component analysis (PCA) by Raman spectroscopic barcode. Similar to our NIR approach, Yang et al. [50] differentiated oils using LDA and CVA with 93% accuracy with their FT-NIR equipment, and Bewig et al. [53] discriminated vegetable oils successfully by NIR reflectance spectroscopy. Based on these previous studies, we explored a novel strategy to apply supervised pattern recognition that allows us to predict the oil type in the further application, and we also analyzed the ability of our model to predict the oil mixture. In addition, to our best knowledge, our study is the first in the literature to apply Raman and NIR to the potato chip (food matrix) oil authentication.

Our model generated by using the Raman and NIR spectra coupled with pattern recognition analysis has adequate ability to rapidly (~1 min for Raman, ~20 sec for NIR) authenticate the mislabeling problem in potato chip products and be a potentially useful tool to perform in-situ screening of potato chip oil types in the market.

### 3.3. PLSR Models for Raman and NIR Spectroscopy

**Figure 5:**
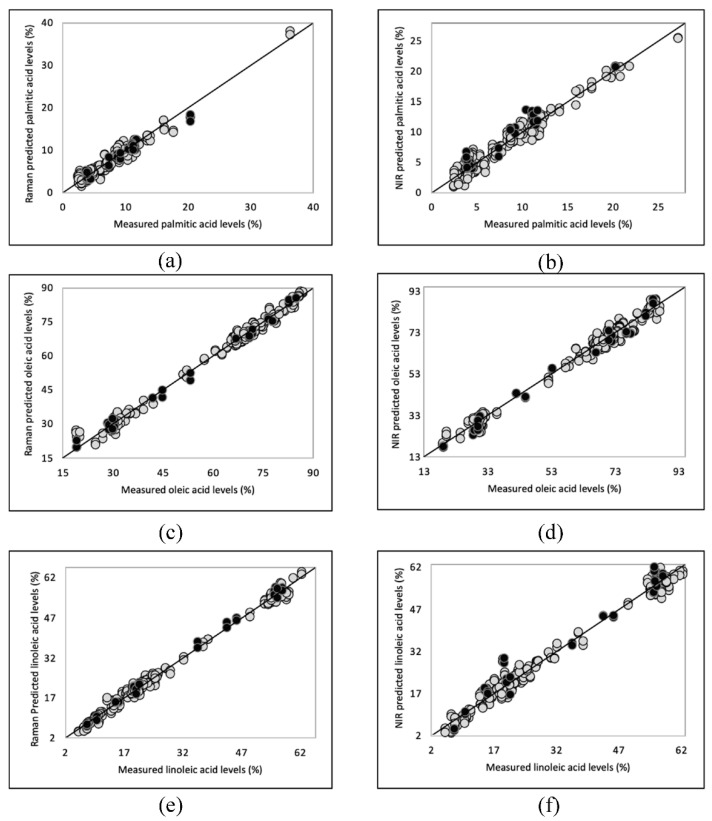
PLSR calibration and validation plots for main fatty acids, palmitic acid (**a**,**b**), oleic acid (**c**,**d**), and linoleic acid (**e**,**f**) in potato chip samples utilizing Raman and NIR data respectively.

Saturated (SFA), monounsaturated (MUFA) and polyunsaturated fatty acids (PUFA), palmitic acid (C16:0), oleic acid (C18:1 n-9) and linoleic acid (C18:2 n-6), respectively, were predominant in vegetable oils and their contents are related to oil and product stability and quality [33]. Therefore, it is crucial to monitor the major fatty acid content in oil during potato chip manufacturing and storage [54]. The quantitative models, partial least squares regression (PLSR) models, were developed using the handheld Raman (1064 nm) and NIR spectral data based on the reference value of fatty acid composition (Figure 5). The performance statistics of PLSR models generated using a calibration (*n* = 102) and external validation (*n* = 16) data set are summarized in Table 4. The number of samples and the range in calibration models are not all the same because of the outlier exclusion [28]. Six factors were chosen to generate all the FTIR and Raman calibration models based on the standard error of cross-validation (leave-one-out) result, achieving the best quality of the models and avoiding the risk of overfitting at the same time [55].

Our PLSR models showed a strong correlation (Rcal > 0.98 and Rval > 0.97) in predicting palmitic, oleic, and linoleic acid content in potato chip oils. The standard error of prediction (SEP) values, ranging from 1.08%–1.84% for the three predominant fatty acids in Raman validation models and ranging from 1.60%–3.55% for NIR external validation models, are similar to the standard error of cross validation (leave-one-out) values in each calibration model which demonstrate the robustness of the models. Overall, the Raman regression models demonstrated superior performance than those generated by the NIR sensor, especially for linoleic acid. The correlation coefficient of validation and SEP for linoleic acid obtained from the Raman model was 1 and 1.31%, respectively. In contrast, the NIR model gave a Rval of 0.99 and a SEP of 3.55%. Our handheld Raman units demonstrated better performance for the prediction of the main fatty acids composition (higher Rcal and Rval) than the study reported by Dong and others (2013) for vegetable oils using a portable Raman spectrometer with a shorter wavelength laser (785 nm) coupled with least squares support vector machines [27]. Meanwhile, our NIR models showed superior performance on higher Rval in predicting oleic and linoleic acids when compared with the past research on oils conducted by Casale et al. [56] and lower SEP in predicting oleic acid compared with the study reported by Sato [57] using their benchtop NIR units.

## 4. Conclusions

This study showed that a handheld Raman device with 1064 nm excitation laser and a miniature NIR sensor allowed for rapid authentication of the oil type used in potato chip manufacturing. Based on the result of GC-FID analysis, a total of 83 (~70%) potato chip samples were identified as having been manufactured with a single oil, including corn oil (19%), canola oil (7%), mid-oleic sunflower oil (12%), high-oleic sunflower (I) (12%), high-oleic sunflower (II) (14%), peanut oil (3%) and cottonseed oil (4%). Combining the pattern recognition analysis, potato chip oils were successfully clustered into their corresponding oil type used in frying and our external validation set demonstrated a 100% accuracy for identifying single oils by using Raman and NIR models. Interestingly, pattern recognition predictions showed that 11% of potato chips (*n* = 13) that indicated a single oil in the label were mislabeled, which was corroborated by GC-FID analysis. In addition, the same spectra allowed the prediction of the major fatty acid composition (palmitic acid, oleic acid and linoleic acid) with strong correlation (Rval > 0.97) and low standard error of prediction. The performance of the PLSR models obtained from the handheld Raman device were superior to models from portable Raman units in other studies and comparable to results from benchtop infrared systems. The handheld Raman spectrometer and miniature NIR sensor can provide applicable tools to perform the rapid authentication of potato chip oil type and in-situ determination of their main fatty acid composition in the market.

## Figures and Tables

**Table 1:** Fatty acid composition summary of oil from potato chip samples and oil references using gas chromatograph flame ionization detector (GC-FID) method.

		Corn	Canola	HO SUN ^a^ (I)	HO SUN ^a^ (II)	MO SUN ^b^	Peanut	Cottonseed
Palmitic (%) C16:0	Range	8.4–14.1	2.9–4.7	2.4–5.2	2.5–4.9	3.5–5.9	3.0–5.0	17.6–21.8
	Mean	11	3.9	4.2	3.9	4.5	4.2	20
	SD	1.4	0.7	0.8	0.7	0.7	0.9	1.6
	Reference oils	9.6	3.9	2.8	——	3.4	8.1	16.8
Stearic (%) C18:0	Range	1.6–2.3	1.9–2.1	2.9–3.8	1.7–4.3	2.1–4.2	2.5–3.3	2.6–3.2
	Mean	1.9	2	3.4	3.2	3.5	2.9	2.9
	SD	0.2	0.1	0.3	0.9	0.8	0.4	0.3
	Reference oils	1.8	1.9	2.6	——	3.5	3.1	2.9
Oleic (%) C18:1 n-9	Range	28.3–32.3	66.6–68.7	82.0–87.1	70.9–78.9	64.1–69.9	75.6–81.4	18.9–20.1
	Mean	30.5	67.6	83.9	74.3	67.6	78.5	19.2
	SD	0.9	0.7	1.7	2.5	1.6	2.6	0.5
	Reference oils	30	66.5	84.3	——	66.6	66.6	20.6
Linoleic (%) C18:2 n-6	Range	54.5–58.5	18.4–19.5	6.7–10.4	14–21.9	22.5–27.6	11.4–15.4	57.0–59.1
	Mean	55.7	19.1	8.4	17.7	24.3	13.8	57.9
	SD	1	0.4	1.2	2.5	1.3	1.7	0.9
	Reference oils	57.6	19.4	10.3	——	26	25.9	59.4
Linolenic (%) C18:3 n-3	Range	0.6–1.0	6.2–9.1	0.0–0.8	0.2–2.4	0.1–1.4	0–0.8	0.0–0.2
	Mean	0.9	7.5	0.2	0.8	0.4	0.4	0.2
	SD	0.1	1.1	0.2	0.8	0.3	0.4	0.1
	Reference oils	1.1	8.4	0	——	0.5	0.4	0.2

^a^ HO SUN: a high-range oleic, above 70% monounsaturated sunflower oil; ^b^ MO SUN: a mid-range oleic, around 65% monounsaturated sunflower oil.

**Table 2:** Interclass distance between 7 types of potato chip frying oil based on the SIMCA training model generated by (**a**) the Raman spectral data collected in the 790–1782 cm^–1^ region and (**b**) NIR spectral data collected in the 1350–2552 nm region.

Groups	HO SUN (I)	MO SUN	Canola	HO SUN (II)	Peanut	Corn	Cottonseed
**(a)**							
HO SUN (I)	0.0						
MO SUN	3.6	0.0					
Canola	7.1	1.5	0.0				
HO SUN (II)	2.0	0.9	3.3	0.0			
Peanut	1.3	3.1	6.5	1.3	0.0		
Corn	10.1	4.5	3.2	5.8	9.7	0.0	
Cottonseed	7.2	3.7	3.0	3.8	7.0	2.6	0.0
**(b)**							
HO SUN (I)	0.0						
MO SUN	3.8	0.0					
Canola	44.8	11.8	0.0				
HO SUN (II)	6.2	2.9	25.3	0.0			
Peanut	8.7	10.5	34.7	7.2	0.0		
Corn	13.0	5.5	15.5	14.5	39.0	0.0	
Cottonseed	40.2	14.5	13.9	26.1	36.1	12.0	0.0

**Table 3:** Specificity and sensitivity values of SIMCA models obtained from the handheld Raman (1064 nm) and the miniature NIR spectral data.

Model Types	Sensitivity (%)	Specificity (%)
Raman	100	50
NIR	100	100

**Table 4:** The performance statistics of Partial Least Square Regression (PLSR) models developed using a training (*n* = 102) and an external validation (*n* = 16) data set based on Raman and NIR spectral data for estimating palmitic, oleic, linoleic acid composition in potato chip samples.

Approach	Fatty Acid	Training Model					External Validation Model			
		Range	*N* ^a^	Factor	SECV ^b^	Rcal	Range	*N* ^c^	SEP ^d^	Rval
**Raman**	Palmitic (%)	2.4–36.3	101	6	1.08	0.98	3.7–20.3	16	1.08	0.97
	Oleic (%)	18.9–86.9	102	6	2.26	1	19.1–84.9	16	1.84	1
	Linoleic (%)	5.3–62.4	102	6	1.48	1	7.5–57.5	16	1.31	1
**NIR**	Palmitic (%)	2.5–27.2	94	6	1.06	0.98	3.7–20.3	16	1.60	0.97
	Oleic (%)	18.9–86.9	95	6	2.61	0.99	19.1–84.9	16	2.87	0.99
	Linoleic (%)	5.3–62.4	101	6	2.47	0.99	7.5–57.5	16	3.55	0.99

^a^ Sample number in the training models. ^b^ Standard error of cross validation. ^c^ Sample number in the external validation models. ^d^ Standard error of prediction.
